# Ion channel classification through machine learning and protein language model embeddings

**DOI:** 10.1515/jib-2023-0047

**Published:** 2024-11-25

**Authors:** Hamed Ghazikhani, Gregory Butler

**Affiliations:** Department of Computer Science and Software Engineering, Concordia University, Montreal, Canada

**Keywords:** ion channels, membrane proteins, transmembrane proteins, drug discovery, protein language models, Convolutional Neural Network

## Abstract

Ion channels are critical membrane proteins that regulate ion flux across cellular membranes, influencing numerous biological functions. The resource-intensive nature of traditional wet lab experiments for ion channel identification has led to an increasing emphasis on computational techniques. This study extends our previous work on protein language models for ion channel prediction, significantly advancing the methodology and performance. We employ a comprehensive array of machine learning algorithms, including k-Nearest Neighbors, Random Forest, Support Vector Machines, and Feed-Forward Neural Networks, alongside a novel Convolutional Neural Network (CNN) approach. These methods leverage fine-tuned embeddings from ProtBERT, ProtBERT-BFD, and MembraneBERT to differentiate ion channels from non-ion channels. Our empirical findings demonstrate that TooT-BERT-CNN-C, which combines features from ProtBERT-BFD and a CNN, substantially surpasses existing benchmarks. On our original dataset, it achieves a Matthews Correlation Coefficient (MCC) of 0.8584 and an accuracy of 98.35 %. More impressively, on a newly curated, larger dataset (DS-Cv2), it attains an MCC of 0.9492 and an ROC AUC of 0.9968 on the independent test set. These results not only highlight the power of integrating protein language models with deep learning for ion channel classification but also underscore the importance of using up-to-date, comprehensive datasets in bioinformatics tasks. Our approach represents a significant advancement in computational methods for ion channel identification, with potential implications for accelerating research in ion channel biology and aiding drug discovery efforts.

## 1 Introduction

**Figure 1:**
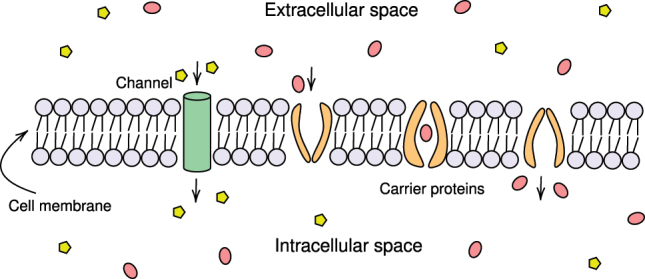
Transmembrane transport proteins. This diagram illustrates two types of transport proteins, channel and carrier proteins.

Ion channels are specialized membrane proteins crucial for regulating ion flux across cellular membranes, thereby controlling cellular electrical properties and a host of physiological functions (Figure 1) 1], [2], [3. They are implicated in myriad biological activities, including but not limited to, muscle contractions, nerve impulse propagation, and intracellular signaling [4]. Given their pivotal role, ion channels have become a focal point in membrane protein research, with comprehensive studies investigating their structural attributes, functional mechanisms, and regulatory dynamics [5, 6]. In the context of drug discovery, ion channels serve as attractive therapeutic targets, as their modulation can induce transformative changes in cellular behavior [7, 8]. Owing to the resource-intensive nature of traditional wet-lab techniques for characterizing ion channels, there has been a burgeoning interest in the development of computational methods as efficient, cost-effective alternatives [9, 10].

A considerable body of research has focused on the computational prediction of ion channel proteins, aiming to distinguish them effectively from non-ion channels 9], [10], [11], [12], [13], [14], [15. Traditional machine learning algorithms like support vector machines (SVM) and random forests (RF) have often been employed, leveraging features extracted from the primary, secondary, and tertiary structures of protein sequences [10, 16]. These features can encompass various sequence-specific attributes such as particular amino acid residues and motifs, as well as structural elements like solvent accessibility [9, 10]. Despite the efficacy of these conventional methods, the advent of deep learning technologies has ushered in new avenues for ion channel prediction [14, 15]. Recent advancements in this domain have illustrated the capability of deep learning models to learn intricate sequence patterns, thereby enhancing the predictive performance and potentially outpacing traditional machine learning paradigms.

Protein language models, which leverage advanced deep learning methods, have increasingly captivated the attention of the computational biology community for their capabilities in faithfully simulating biological systems [3, 17], [18], [19], [20], [21], [22], [23. These models offer robust and nuanced representations of protein sequences, which find applications in a diverse array of protein analyses, such as functional annotation, elucidating protein-protein interactions, and structural prediction 17], [18], [19], [20], [21, 24, 25]. A comprehensive review by Unsal et al. chronicles the evolution and application of these natural language models in the domain of protein science from 2015 to the present [26].

Utilizing deep learning architectures, protein language models can be pre-trained on extensive protein sequence databases and subsequently fine-tuned for specialized tasks [26]. This modus operandi, referred to as transfer learning [27], enables the models to apply the foundational knowledge gained during the pre-training phase to excel in the targeted analytical tasks. Pre-training on a comprehensive and diverse dataset imbues these models with the ability to discern universal patterns in protein sequences, offering broad applicability across various computational challenges [20]. Subsequent fine-tuning on task-specific datasets allows these models to hone their capabilities, resulting in optimized performance for the designated task. This methodology has proven efficacious in diverse applications within protein analysis, including but not limited to, functional prediction [28], deciphering protein-protein interactions [29], and structural determination [30, 31].

Within the scope of the ProtTrans initiative [20], Elnaggar et al. undertook a rigorous evaluation of six Transformer-based models across a variety of analytical tasks, such as secondary structure prediction, subcellular localization, and water solubility assessments. Included in this evaluation were ProtBERT and ProtBERT-BFD, two BERT-derived models pre-trained on expansive protein databases: UniRef100, containing 216 million protein sequences [32], and the BFD database, boasting 2.1 billion sequences [33]. These models each comprise 30 layers with 16 attention head transformer encoders, totaling 420 million parameters. They are designed to output 1024-dimensional vectors for individual amino acids within a protein sequence. Further, MembraneBERT [34] is an advanced protein language model that was fine-tuned from ProtBERT-BFD, utilizing a dataset of membrane proteins curated from the TooT-M project [35].

In our preceding research, we unveiled TooT-BERT-T [36] and TooT-BERT-C [37], methodologies designed to distinguish between transport proteins and non-transport proteins, as well as ion channels and non-ion channels. These methods employ a Logistic Regression (LR) classifier and leverage fine-tuned representations extracted from ProtBERT-BFD. Notably, our approaches surpassed existing benchmarks in both transporter and ion channel classification, underscoring the effectiveness of incorporating protein language models into these predictive tasks.

In the current study, we significantly advance the state-of-the-art in ion channel prediction by introducing a novel approach that combines the power of protein language models with Convolutional Neural Networks (CNNs). This innovative fusion represents a significant leap forward in the field, as it leverages the contextual understanding of protein sequences provided by language models with the hierarchical feature extraction capabilities of CNNs. While protein language models have shown promise in capturing long-range dependencies and semantic information in protein sequences, and CNNs have demonstrated effectiveness in identifying local motifs and spatial patterns, their combination for ion channel classification remains largely unexplored. Our approach, TooT-BERT-CNN-C, uniquely harnesses the strengths of both techniques, allowing for a more nuanced and comprehensive analysis of protein sequences. This synergy enables our model to simultaneously consider both the global context of the entire protein sequence and the local, structurally important features that are crucial for ion channel function. By bridging these two powerful methodologies, we create a more robust and accurate classification system that pushes the boundaries of computational protein analysis.

In the present study, we offer the following key contributions:

1. We explore the effectiveness of fine-tuned embeddings from ProtBERT, ProtBERT-BFD, and MembraneBERT in the context of ion channel prediction, shedding light on their applicability for this specific task.
2. We conduct a comprehensive evaluation of various classical machine learning classifiers, such as SVM, RF, kNN, FFNN, and LR, as well as a CNN, all employing these fine-tuned representations for ion channel discrimination.
3. We introduce a novel CNN architecture tailored for fine-tuning the embeddings from ProtBERT, ProtBERT-BFD, and MembraneBERT, which excels in differentiating ion channels from non-ion channels.
4. We present TooT-BERT-CNN-C, a cutting-edge methodology for ion channel prediction that combines fine-tuned embeddings from ProtBERT-BFD and a CNN, thereby surpassing existing state-of-the-art methods.

The remainder of this paper unfolds as follows: Section 2 delineates the datasets and methodologies implemented in our experiments, elaborating on the protein language models, traditional machine learning classifiers, and our newly designed CNN architecture. Section 3 showcases the experimental results, offering a performance comparison of the various classifiers in ion channel prediction, along with a discussion on the significance and implications of our findings. The paper concludes with Section 4, where we summarize our key contributions and propose avenues for future research.

## 2 Materials and methods

### 2.1 Dataset

Our study utilizes two datasets: the original DS-C used in previous works and a newly curated DS-Cv2, which significantly expands upon and updates DS-C.

DS-C, sourced from the UniProt database, comprises 301 ion channels and 4,263 non-ion channels, totaling 4,564 sequences. This dataset, used in prior works like DeepIon [15] and MFPS_CNN [14], was curated using the BLAST algorithm to exclude protein sequences with over 20 % similarity. Table 1 details the distribution of sequences in DS-C.

**Table 1:** DS-C: distribution of sequences in the original ion channel dataset.

Class	Training	Test	Total
Ion channel	241	60	301
Non-ion channel	3,413	850	4,263
Total	3,654	910	4,564

To address the need for more comprehensive and current data, we curated DS-Cv2 on February 11, 2024, from UniProtKB/Swiss-Prot entries. The curation process involved:

1. Using CD-HIT to remove sequences with over 20 % similarity.
2. Focusing exclusively on ion channels and other membrane proteins, excluding ion transporters.
3. Partitioning the dataset into training and test sets following an 80:20 ratio.

DS-Cv2 comprises 525 ion channels and 11,130 other membrane proteins, a substantial increase from DS-C. Table 2 details the distribution of sequences in DS-Cv2.

**Table 2:** DS-Cv2: distribution of sequences in the updated ion channel dataset.

Class	Training	Test	Total
Ion channel (IC)	420	105	525
Other membrane protein (MP)	8,904	2,226	11,130
Total	9,324	2,331	11,655

By utilizing both datasets, we demonstrate our approach’s robustness across different data distributions and sizes while providing a direct comparison with previous methodologies.

### 2.2 Protein language models

**Figure 2:**
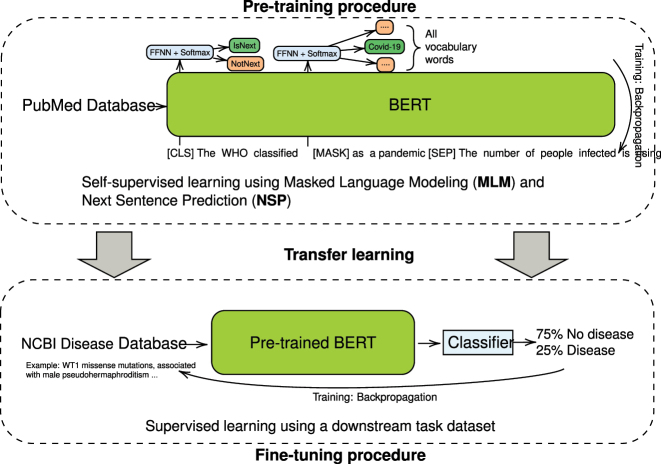
BERT training stages. The BERT model training process is depicted in this figure, with the pre-training phase involving the use of Masked Language Modeling (MLM) and Next Sentence Prediction (NSP) objective to learn contextual representations of words from the PubMed Database. The fine-tuning phase involves adapting the pre-trained BERT model to the binary classification task of predicting whether a text from the NCBI Disease Database describes a disease or does not describe a disease.

Transfer learning is a technique that allows a model to use knowledge gained from one task to improve its performance on a related, but different task [38]. This can be particularly useful when there is a limited amount of labeled data available for the target task, as the model can utilize the knowledge gained from the source task to enhance its performance. The process of transfer learning consists of two steps: Pre-training and fine-tuning (see Figure 2).

Pre-training involves training a model on a large, general-purpose dataset, allowing the model to learn general patterns and representations that are applicable to a variety of tasks [39]. Fine-tuning involves further training the pre-trained model on a specific task or dataset, allowing the model to adapt to the characteristics of that task or dataset and often resulting in improved performance [39].

BERT [27], or Bidirectional Encoder Representations from Transformers, is a transformer-based [40] language model that has achieved state-of-the-art performance on a variety of natural language processing tasks. BERT is able to generate contextualized representations of words in a sentence, taking into account the words that come before and after it [41]. This is achieved through the use of self-attention mechanisms [40], which allow the model to attend to different parts of the input sequence and weight their importance for generating the final representation.

BERT (Figure 2) has a multi-layered architecture of self-attention and feed-forward networks in each layer and is trained using two pre-training tasks: Masked Language Modeling (MLM) and Next Sentence Prediction (NSP). In the MLM task, a percentage of the tokens in the input are randomly masked and the model is required to predict the original value of the masked tokens based on the context provided by the remaining tokens. The NSP task involves predicting whether a given sentence is the next sentence in a sequence or a randomly chosen sentence from the dataset. By performing these pre-training tasks, BERT is able to learn contextual relationships between words in a sentence and between sentences in a document. Once pre-trained, BERT can be fine-tuned for specific classification tasks by adding a classification head on top of the model.

Frozen (feature-based) and fine-tuned representations are two different types of representations that can be extracted from a BERT model. Frozen representations, are derived from the internal layers of a BERT model that has been pre-trained on a large, general-purpose dataset. These representations capture general patterns and structures in the data that are useful for a wide range of tasks. Fine-tuned representations, on the other hand, are derived from a BERT model that has been further trained on a specific task or dataset. These representations are tailored to the specific characteristics of the task or dataset, and can often provide better performance for that task or dataset compared to feature-based representations.

In self-attention, a sequence of *n* items is transformed into a sequence of *n* vectors. The *i*th vector is computed by applying the self-attention mechanism to the *i*th item and all other items in the sequence. The self-attention mechanism is defined by three functions: the query function *Q*, the key function *K*, and the value function *V*. The value function *V* is typically implemented as a linear transformation of the input item, such as a matrix multiplication followed by an optional non-linear activation function. Given an input sequence of items *x*_1_, *x*_2_, …, *x*_*n*_, the self-attention mechanism produces an output sequence of vectors *y*_1_, *y*_2_, …, *y*_*n*_ with the following formula:

(1) $$y_{i}=\overset{n}{\underset{j=1}{\sum }}a_{i,j}V\left(x_{j}\right)$$

where *a*_*i*,*j*_ is a weight that indicates the importance of item *x*_*j*_ to the computation of vector *y*_*i*_. These weights are computed by taking the dot product of the query vector *Q*(*x*_*i*_) and the key vector *K*(*x*_*j*_) for all pairs of items *i* and *j*, and then normalizing these dot products using the softmax function:

(2) $$a_{i,j}=\frac{\exp\left(Q\left(x_{i}\right)\cdot K\left(x_{j}\right)\right)}{\overset{n}{\underset{k=1}{\sum }}\exp\left(Q\left(x_{i}\right)\cdot K\left(x_{k}\right)\right)}$$

Protein language models, a subclass of natural language models, have gained significant attention in recent years due to their ability to generate highly expressive and informative representations of protein sequences. These representations have been widely applied to various tasks in computational biology, including predicting protein function, structure, and interactions [18, 20, 24, 26, 42, 43].

ProtBERT [20] is a protein language model that was pre-trained on the UniRef100 database [44], which contains over 216 million protein sequences. It is based on the BERT model architecture [27] and uses the MLM pre-training task. ProtBERT-BFD [20] is a variant of ProtBERT that was pre-trained on the BFD database [33], which consists of over 2.1 billion protein sequences. MembraneBERT [34] is a variant of ProtBERT-BFD that has been fine-tuned on a dataset of membrane proteins [35], enabling it to specifically focus on the characteristics of these proteins.

### 2.3 Machine learning classifiers

In this study, we employ various classical machine learning classifiers and a convolutional neural network (CNN) to distinguish ion channels from non-ion channels using representations generated by ProtBERT, ProtBERT-BFD, and MembraneBERT. We optimize each classifier’s performance through grid search over a set of hyperparameters on the training set (see Table 3).

**Table 3:** Optimal hyperparameters of classifiers. The table lists the optimal hyperparameters of the classifiers determined through grid search.

Classifier	Selected parameters
kNN	Algorithm: auto, leaf size: 10, number of neighbors: 5
RF	Min samples leaf: 1, min samples split: 2, number of estimators: 100
SVM	C: 10, gamma: 0.1, kernel: rbf
LR	C: 1, max iteration: 100, solver: lbfgs
FFNN	Alpha: 0.0001, hidden layer sizes: (256, 32), learning rate: invscaling
CNN	# epochs: 10, learning rate: 5 × 10^−5^, batch size: 4

#### 2.3.1 Classical machine learning classifiers

In our study, we employ a diverse set of classical machine learning classifiers from the scikit-learn library [45], each chosen for its proven effectiveness in various bioinformatics tasks and ability to capture different aspects of the data. Logistic Regression (LR) [46] is utilized for its capacity to estimate class probabilities based on linear combinations of input features. We also implement Support Vector Machine (SVM) [47], which excels in finding an optimal hyperplane to separate classes in a high-dimensional space. The Random Forest (RF) classifier [48] is included as an ensemble method, combining predictions from multiple decision trees through majority voting. For instance-based learning, we employ the k-Nearest Neighbor (kNN) algorithm [49], which classifies based on the majority class of k nearest neighbors in the feature space. Lastly, we incorporate a Feed-Forward Neural Network (FFNN) [50, 51], a multi-layer perceptron capable of learning complex non-linear relationships in the data. This comprehensive selection of classifiers allows us to thoroughly evaluate and compare different machine learning approaches in the context of ion channel prediction.

#### 2.3.2 Convolutional Neural Network (CNN)

We implement a CNN using PyTorch [52] to classify ion channels and non-ion channels, as well as to fine-tune ProtBERT, ProtBERT-BFD, and MembraneBERT concurrently during training. The CNN architecture consists of multiple convolutional layers, a dropout layer for regularization [53], and three fully connected layers. This architecture is well-suited for processing sequential data like protein sequences [54, 55].

### 2.4 Proposed method

**Figure 3:**
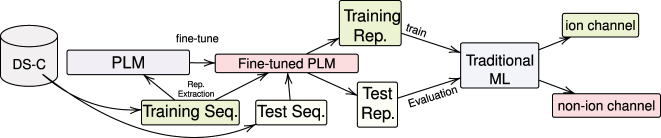
Proposed method of using PLMs and traditional classifiers. Schematic representation of the proposed method for ion channel classification, which combines protein language models (PLMs) such as ProtBERT, ProtBERT-BFD, and MembraneBERT with traditional machine learning classifiers to distinguish ion channels from non-ion channels. The process entails fine-tuning the BERT-based models using the training and validation sets and subsequently extracting representations from the training and test sets to assess the performance of traditional classifiers, including kNN, RF, LR, SVM, and FFNN.

**Figure 4:**
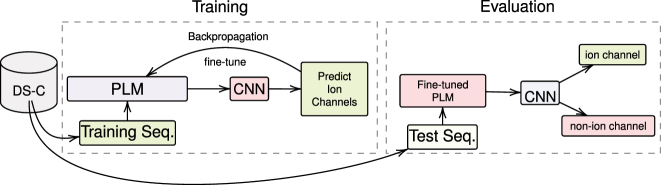
Proposed method for ion channel classification using CNN. This figure illustrates the proposed methodology for distinguishing ion channels from non-ion channels using a deep learning classifier, CNN. The process entails the concurrent training of CNN and fine-tuning of protein language models (PLMs), which include ProtBERT, ProtBERT-BFD, and MembraneBERT.

Our proposed method for protein classification is illustrated in Figure 3. It involves using both traditional machine learning algorithms (Figure 3) and a deep learning-based approach (Figure 4) to classify ion channel proteins from non-ion channel membrane proteins.

For classical machine learning classifiers, the BERT-based models are first fine-tuned using a one-layer feed-forward classifier. The learned representations are then extracted and used as input for the classifiers.

For the CNN-based approach, the BERT models are fine-tuned concurrently with the CNN during training, allowing the models to learn task-specific representations. These representations are then used as input for the CNN to classify the proteins.

During training, the classifiers are trained on the training and validation sets (10 % of the training data), which are used to fine-tune the BERT-based models. The models are then evaluated on the test set to assess their generalization ability on unseen data.

### 2.5 Grid search

In this work, we used the grid search method to find the optimal hyperparameter values for each classifier (see Table 3). The grid search method involves training and evaluating a model on a grid of hyperparameter values, using cross-validation (CV) to estimate the model’s performance, in order to find the combination of hyperparameter values that results in the best performance on the data. By applying the grid search method, we were able to identify the classifier and hyperparameter combination that achieved the best performance on the data.

### 2.6 Evaluation methods

In our study, we used 5-fold CV to evaluate the performance of all of the classifiers. CV is a technique that involves dividing the dataset into a number of folds, training the model on some of the folds, and evaluating the model on the remaining folds. This process is repeated a number of times, with different combinations of training and evaluation folds, in order to obtain a more robust estimate of the model’s performance. The optimal hyperparameters for each classifier were determined based on the results of the CV, and are shown in Table 3.

#### 2.6.1 McNemar’s test

We utilize McNemar’s test to evaluate the differences in performance between our proposed method, TooT-BERT-CNN-C, and the state-of-the-art model TooT-BERT-C. McNemar’s test [56] is a statistical test used to compare the performance of two different classifiers on a binary classification task. It is typically used when the same set of samples has been classified by both classifiers, and the goal is to determine whether the performance of one classifier is significantly better than the other.

The test is based on the contingency table, which is a 2 × 2 table that compares the outcomes of two classification methods. The formula for McNemar’s test is:

(3) $$\chi^{2}=\frac{{\left(b-c\right)}^{2}}{b+c}$$

where *b* represents the number of cases where the first model made an incorrect prediction, while the second model made a correct prediction. *c* represents the number of cases where the first model made a correct prediction, while the second model made an incorrect prediction.

To interpret the results of McNemar’s test, a *p*-value is calculated based on the chi-square statistic. If the *p*-value is less than a predetermined threshold (usually 0.05), then it is considered statistically significant and the null hypothesis (that there is no difference in performance between the two classifiers) is rejected. This indicates that there is a statistically significant difference in the performance of the two classifiers.

#### 2.6.2 Evaluation metrics

For this paper, we used a variety of performance metrics to evaluate the effectiveness of our approach for predicting ion channels. These metrics included accuracy (Acc), sensitivity (Sen), specificity (Spc), and the Matthew’s correlation coefficient (MCC).

The dataset used in this work, consists of 301 ion channels and 4,263 non ion-channels, which is an imbalanced dataset. This means that the number of samples in each class is not equal, and this can affect the performance of machine learning algorithms. In imbalanced datasets, accuracy can be misleading as it does not take into account the relative frequencies of the different classes. This can lead to the model achieving high accuracy by simply predicting the majority class all the time, even if it has poor performance on the minority class. Therefore, it is often recommended to use metrics that consider all classes, such as the MCC, which takes into account true and false positives and negatives [57].

Accuracy is a measure of the overall correct classification rate and is calculated as the number of correct predictions divided by the total number of predictions. It is expressed as a percentage and can be calculated using the following formula:

(4) $$Accuracy=\frac{TP+TN}{TP+TN+FP+FN}$$

Sensitivity, also known as the true positive rate, is a measure of the proportion of actual positive cases that are correctly identified as such. It is calculated using the following formula:

(5) $$Sensitivity=\frac{TP}{TP+FN}$$

Specificity, also known as the true negative rate, is a measure of the proportion of actual negative cases that are correctly identified as such. It is calculated using the following formula:

(6) $$Speci\mathrm{fi}city=\frac{TN}{TN+FP}$$

MCC is a measure of the overall accuracy of a binary classifier, taking into account both the true and false positive and negative rates. It can range from −1 (perfectly incorrect) to 1 (perfectly correct) and is calculated using the following formula:

(7) $$MCC=\frac{TP\cdot TN-FP\cdot FN}{\sqrt{\left(TP+FP\right)\left(TP+FN\right)\left(TN+FP\right)\left(TN+FN\right)}}$$

where TP (True Positive) is a case where the classifier correctly predicts the positive class, TN (True Negative) is a case where the classifier correctly predicts the negative class, FP (False Positive) is a case where the classifier incorrectly predicts the positive class, and FN (False Negative) is a case where the classifier incorrectly predicts the negative class.

## 3 Results and discussion

### 3.1 Protein sequence evaluation

A primary challenge in employing protein language models is the constraint of fixed sequence lengths, which may lead to the omission of crucial structural or functional details. In our methodology, we limited the protein sequences to a length of 1,024 due to computational constraints while fine-tuning ProtBERT, ProtBERT-BFD, and MembraneBERT. For this, we utilized the tokenizer from the Transformers Python library, setting a maximum length parameter of 1,024 and enabling automatic truncation. This approach ensures that any sequence exceeding this limit is truncated, while retaining the first 1,024 amino acids for analysis.

**Figure 5:**
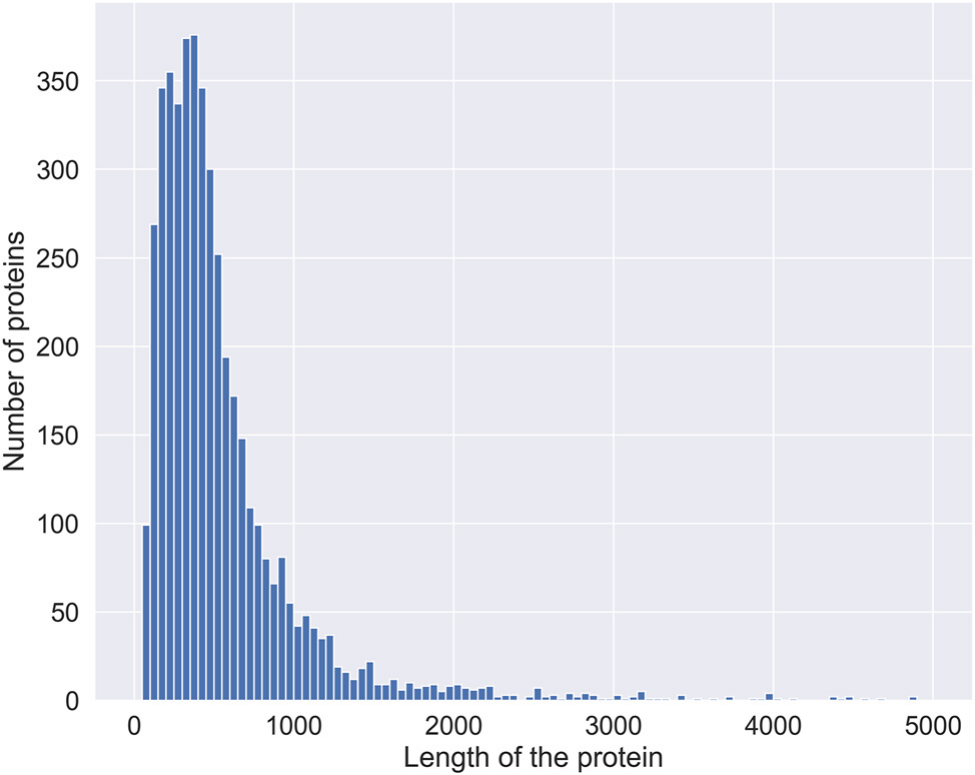
Distribution of protein lengths in the dataset. The distribution of protein lengths in the dataset is depicted in this histogram, with the *x*-axis representing the length of the proteins and the *y*-axis indicating the number of proteins.

We conducted an analysis, depicted in Figure 5, to evaluate the implications of this truncation on our dataset. The histogram demonstrates the range of protein sequence lengths in the dataset. Interestingly, most of the sequences fall below the truncation limit of 1,024 amino acids, suggesting that the truncation during fine-tuning of the BERT models is unlikely to result in substantial information loss.

To visualize the feature representations from ProtBERT, ProtBERT-BFD, and MembraneBERT for ion channels and non-ion channels, we employ t-SNE, or t-Distributed Stochastic Neighbor Embedding, as outlined by van der Maaten et al. ([58]). This technique serves as a powerful tool for reducing dimensionality while maintaining the relationships among high-dimensional data points. This approach is particularly useful for capturing intricate, non-linear relationships, making it widely used in areas like machine learning and data visualization.

**Figure 6:**
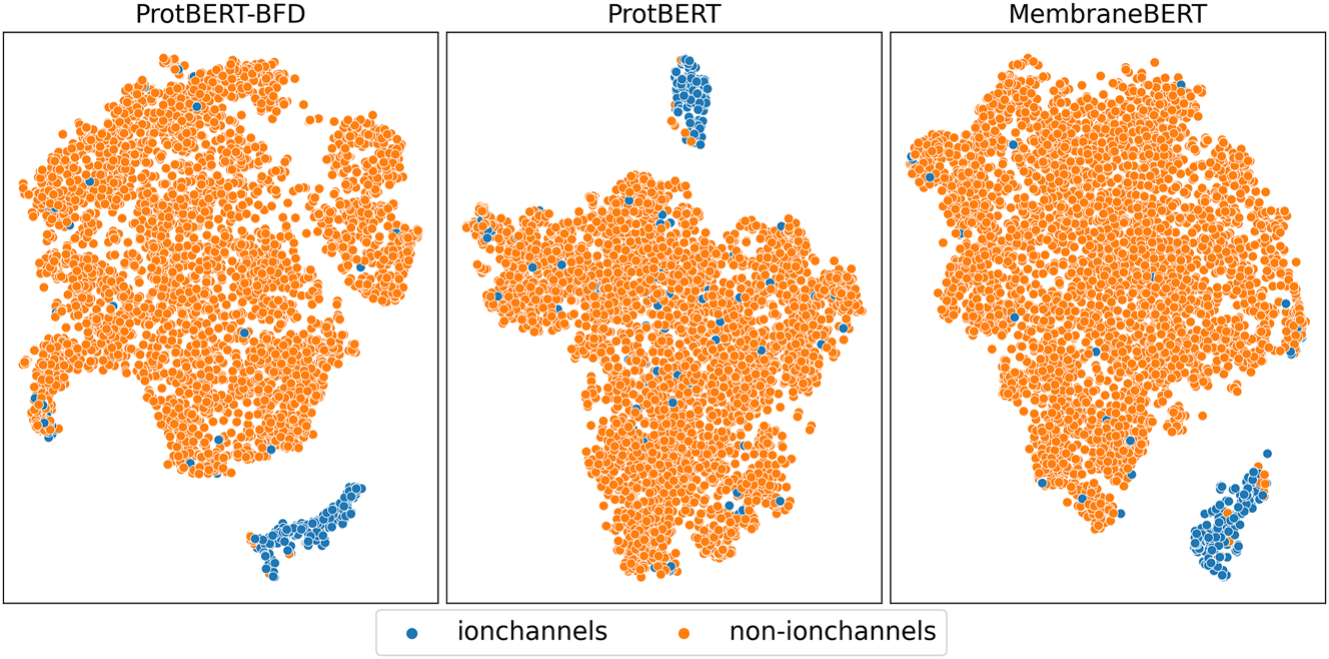
t-SNE plot of BERT representations. A t-SNE plot is shown in this figure, illustrating the two-dimensional representations of the sequences obtained from ProtBERT, ProtBERT-BFD, and MembraneBERT for the ion channel and non-ion channel classes in the dataset. The ion channel sequences are plotted in blue, while the non-ion channel sequences are plotted in orange.

The t-SNE plot, as shown in figure Figure 6, highlights the proficiency of ProtBERT, ProtBERT-BFD, and MembraneBERT in differentiating important features of ion channels from those of non-ion channels. In the plot, ion channels are marked in blue and non-ion channels in orange. The separation between the two categories in this two-dimensional representation suggests that the models effectively capture essential distinctions between the two groups. These distinctions may encompass variations in sequence composition or structural features that serve as hallmarks for ion channel proteins.

### 3.2 Execution time analysis

In this section, we assess the computational overhead associated with fine-tuning ProtBERT-BFD, contrasting setups with and without the inclusion of CNN layers. Although incorporating CNN layers typically escalates both computational demands and execution time, our empirical data suggest that the improvements in model performance justify this additional investment.

Specifically, fine-tuning ProtBERT-BFD without CNN integration took approximately 2 h when executed on a Tesla V100 GPU equipped with 32 GB RAM. The incorporation of CNN layers extended this time frame to roughly 2 h and 30 min. This increment in training duration can be directly attributed to the computational complexity introduced by the added CNN layers.

### 3.3 Comparative analysis of classifier and PLM performances

**Figure 7:**
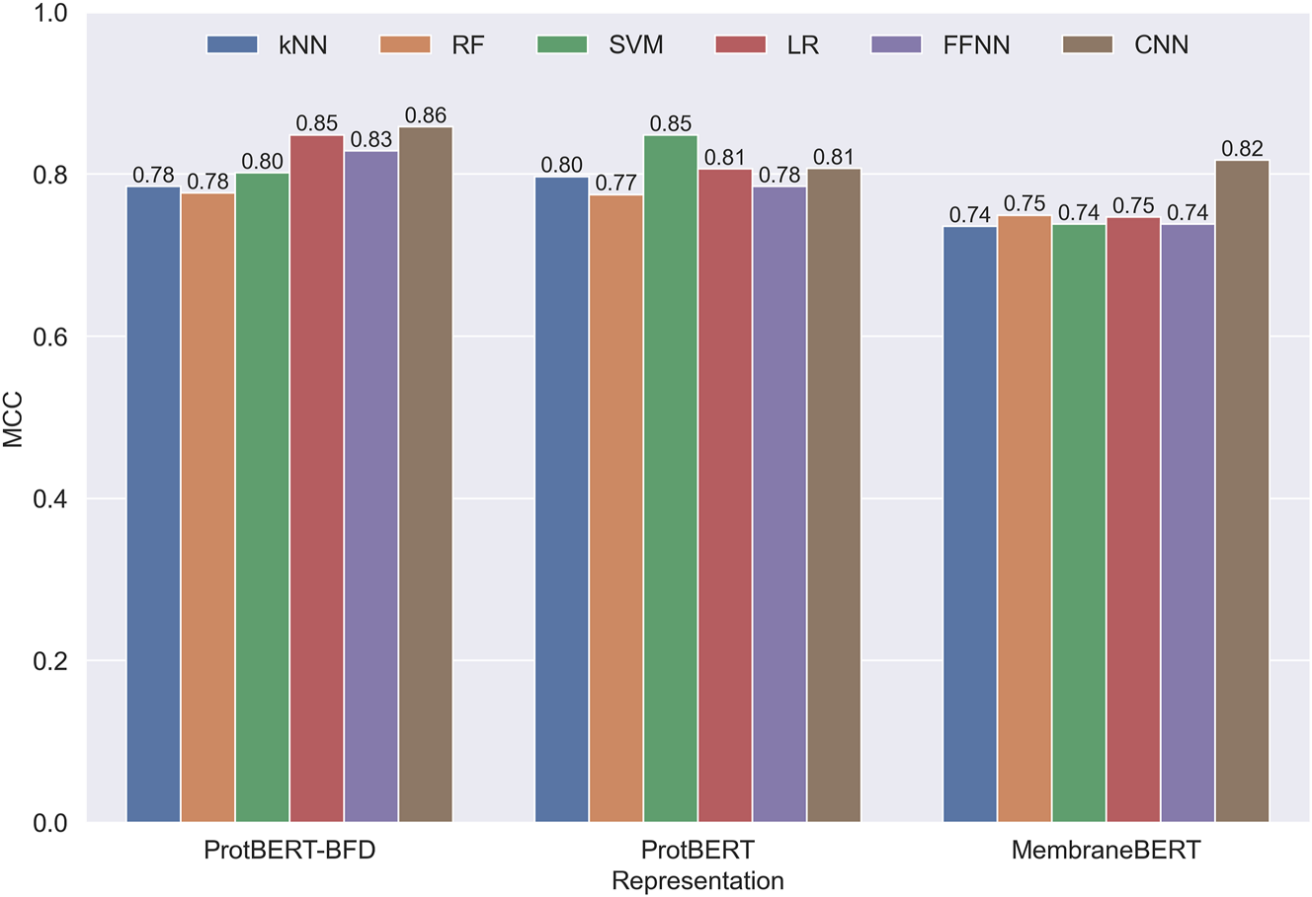
Performance comparison of different classifiers. The performance of different classical and deep learning classifiers and representations generated from ProtBERT, ProtBERT-BFD and MembraneBERT is compared on independent test sets using the MCC metric in this figure.

This section provides a comprehensive analysis of the performance metrics for various Protein Language Models (PLMs) and classifiers. The evaluation metrics include Accuracy, Sensitivity, Specificity, and the Matthews Correlation Coefficient (MCC). Results from both cross-validation (CV) and independent tests are presented in Table 4 and visualized in Figure 7.

**Table 4:** Performance comparison of different classifiers and representations. The table presents a comparison of the performance of different classical and deep learning classifiers and representations, as generated from ProtBERT, ProtBERT-BFD, and MembraneBERT on CV and independent test sets. The results are evaluated using various metrics, with the largest value in each column on independent test results indicated in boldface for comparison between different classifiers. The second best result in each column is indicated with an underline for further analysis and comparison.

PLM	Method	Acc (%)		Sen (%)		Spc (%)		MCC	
		CV	Ind.	CV	Ind.	CV	Ind.	CV	Ind.
ProtBERT	kNN	97.80 ± 0.23	97.69	71.61 ± 3.75	70.00	99.65 ± 0.25	99.53	0.8086 ± 0.0213	0.7972
	RF	97.30 ± 0.22	97.58	60.69 ± 3.14	63.33	99.88 ± 0.15	**100.00**	0.7557 ± 0.0234	0.7749
	SVM	95.22 ± 0.27	98.24	37.63 ± 4.23	75.00	99.29 ± 0.23	**100.00**	0.4469 ± 0.0358	0.8483
	LR	97.57 ± 0.25	97.80	67.13 ± 3.77	68.33	99.72 ± 0.25	**100.00**	0.7852 ± 0.0234	0.8068
	FFNN	97.13 ± 0.54	98.02	67.04 ± 4.23	73.33	98.69 ± 1.42	**100.00**	0.7126 ± 0.0299	0.7848
	CNN	99.09 ± 0.82	97.80	88.90 ± 3.82	66.66	99.82 ± 0.21	**100.00**	0.9235 ± 0.0728	0.8070
Average ProtBERT			97.86		69.44		99.92		0.8032
ProtBERT-BFD	kNN	98.97 ± 0.31	97.47	88.19 ± 5.12	75.00	99.73 ± 0.10	98.71	0.9137 ± 0.0275	0.7848
	RF	99.03 ± 0.45	97.47	87.97 ± 5.85	**76.67**	99.80 ± 0.12	98.82	0.9187 ± 0.0390	0.7767
	SVM	98.39 ± 0.40	97.69	79.87 ± 5.49	75.00	99.69 ± 0.13	**100.00**	0.8450 ± 0.0353	0.8016
	LR	98.96 ± 0.43	98.24	86.71 ± 5.55	**76.67**	99.82 ± 0.11	99.76	0.9123 ± 0.0375	0.8486
	FFNN	98.34 ± 0.92	97.03	82.08 ± 7.42	**76.67**	99.38 ± 0.78	**100.00**	0.8557 ± 0.0584	0.8287
	CNN	99.39 ± 0.20	**98.35**	93.38 ± 2.96	75.00	99.82 ± 0.14	**100.00**	0.9506 ± 0.0167	**0.8584**
Average ProtBERT-BFD			97.71		75.84		99.55		0.8165
MembraneBERT	kNN	99.59 ± 0.34	96.92	96.48 ± 3.03	66.67	99.81 ± 0.18	98.82	0.9665 ± 0.0278	0.7358
	RF	99.59 ± 0.33	97.03	96.32 ± 2.84	68.33	99.82 ± 0.17	98.94	0.9670 ± 0.0274	0.7495
	SVM	99.29 ± 0.32	97.03	91.86 ± 3.13	68.33	99.81 ± 0.15	**100.00**	0.9397 ± 0.0264	0.7383
	LR	99.50 ± 0.33	97.14	95.53 ± 2.88	66.67	99.78 ± 0.17	99.29	0.9590 ± 0.0275	0.7472
	FFNN	99.18 ± 0.44	97.25	91.07 ± 4.84	68.33	99.77 ± 0.20	**100.00**	0.9159 ± 0.0491	0.7383
	CNN	97.86 ± 1.57	97.91	75.55 ± 4.55	68.33	99.44 ± 1.04	**100.00**	0.8203 ± 0.1267	0.8175
Average MembraneBERT			97.21		67.78		99.51		0.7544

#### 3.3.1 Overall performance

ProtBERT-BFD emerges as the strongest performer, particularly in Sensitivity, with an average of 75.84 % on independent test sets. This demonstrates its superior ability to identify true positive cases, crucial in bioinformatics applications. In contrast, MembraneBERT shows high Sensitivity in CV tests but lower performance on independent test sets, suggesting potential overfitting issues.

#### 3.3.2 Classifier performance

The kNN algorithm performs exceptionally well when paired with ProtBERT and MembraneBERT, especially in terms of Sensitivity. This suggests that kNN’s instance-based learning approach synergizes well with these PLM’s feature representations. The CNN, when used with ProtBERT-BFD, achieves the highest MCC (0.8584) on independent test sets, indicating a well-balanced performance across classes.

#### 3.3.3 Stability and consistency

The kNN classifier demonstrates the most stable performance across different PLMs, exhibiting the least variance in CV metrics. This stability makes kNN a robust choice for protein classification tasks. Conversely, MembraneBERT shows significant inconsistencies between CV and independent test set performances, particularly in Sensitivity, warranting further investigation into its generalization capabilities.

#### 3.3.4 Best individual performance

The combination of ProtBERT with SVM achieves the highest accuracy (98.24 %) and MCC (0.8483) among independent tests, suggesting this pairing as a promising configuration for further exploration in protein classification tasks.

### 3.4 Performance analysis on updated dataset (DS-Cv2)

To further validate our approach and assess its generalization capabilities, we evaluated our models on the newly curated dataset, DS-Cv2. Table 5 presents the performance metrics for various classifiers, including CNN, and protein language models on this updated dataset.

**Table 5:** Performance comparison of classifiers and representations on DS-Cv2. Results are presented for cross-validation (CV) and independent test sets (Ind.) using ProtBERT, ProtBERT-BFD, and MembraneBERT. The best result for each metric is highlighted in boldface.

PLM	Method	Acc (%)		Sen (%)		Spc (%)		MCC		ROC AUC	
		CV	Ind.	CV	Ind.	CV	Ind.	CV	Ind.	CV	Ind.
ProtBERT	kNN	98.35 ± 0.08	98.71	99.49 ± 0.07	99.55	74.38 ± 2.56	99.55	0.7977 ± 0.0118	0.8444	0.9391 ± 0.0092	0.9448
	RF	98.39 ± 0.05	98.37	99.65 ± 0.02	99.64	71.57 ± 0.87	99.64	0.7979 ± 0.0066	0.7954	0.9740 ± 0.0013	0.9713
	SVM	97.60 ± 0.74	98.46	99.03 ± 0.73	99.60	67.29 ± 5.96	99.60	0.7152 ± 0.0737	0.8084	0.8956 ± 0.0601	0.9816
	LR	98.28 ± 0.04	98.54	99.43 ± 0.17	99.37	73.89 ± 2.78	99.37	0.7893 ± 0.0034	0.8261	0.9573 ± 0.0049	0.9610
	FFNN	98.36 ± 0.08	98.46	99.39 ± 0.10	99.55	76.47 ± 1.11	99.55	0.8016 ± 0.0088	0.8094	0.9725 ± 0.0023	0.9765
	CNN	**99.23** **±** **0.48**	99.27	**84.05** **±** **11.43**	84.76	**99.94** **±** **0.06**	99.96	**0.9049** **±** **0.0616**	**0.9120**	**0.9945** **±** **0.0063**	0.9903
ProtBERT-BFD	kNN	98.73 ± 0.04	98.71	99.76 ± 0.08	99.91	76.90 ± 1.20	99.91	0.8428 ± 0.0039	0.8395	0.9479 ± 0.0076	0.9485
	RF	98.80 ± 0.03	98.67	99.80 ± 0.01	99.91	77.70 ± 0.65	99.91	0.8521 ± 0.0041	0.8337	0.9757 ± 0.0018	0.9825
	SVM	95.61 ± 2.55	98.71	98.15 ± 1.76	99.91	41.94 ± 30.66	99.91	0.4398 ± 0.3207	0.8395	0.8788 ± 0.0953	0.9749
	LR	98.68 ± 0.15	98.71	99.52 ± 0.19	99.82	80.82 ± 0.96	99.82	0.8416 ± 0.0153	0.8401	0.9761 ± 0.0064	0.9699
	FFNN	98.55 ± 0.07	98.71	99.44 ± 0.09	99.73	79.56 ± 1.31	99.73	0.8258 ± 0.0081	0.8411	0.9775 ± 0.0021	0.9735
	CNN	99.10 ± 0.48	**99.57**	80.71 ± 11.20	**92.38**	**99.97** **±** **0.07**	99.91	0.8885 ± 0.0622	**0.9492**	0.9911 ± 0.0101	**0.9968**
MembraneBERT	kNN	98.90 ± 0.03	98.54	99.60 ± 0.05	99.51	84.14 ± 0.79	99.51	0.8681 ± 0.0030	0.8223	0.9727 ± 0.0042	0.9588
	RF	99.03 ± 0.02	98.67	99.71 ± 0.01	99.64	84.51 ± 0.32	99.64	0.8824 ± 0.0024	0.8368	0.9883 ± 0.0013	0.9792
	SVM	94.46 ± 3.24	98.88	97.73 ± 2.13	99.51	25.15 ± 35.41	99.51	0.2550 ± 0.3907	0.8681	0.8871 ± 0.1479	0.9817
	LR	**99.06** **±** **0.07**	98.76	**99.62** **±** **0.07**	99.42	**87.18** **±** **0.63**	99.42	**0.8889** **±** **0.0077**	0.8535	0.9812 ± 0.0055	0.9663
	FFNN	99.01 ± 0.07	98.76	99.60 ± 0.09	99.37	86.61 ± 1.70	99.37	0.8833 ± 0.0079	0.8547	**0.9918** **±** **0.0017**	0.9853
	CNN	98.67 ± 0.42	98.16	80.71 ± 10.58	59.05	99.52 ± 0.69	**100.00**	0.8464 ± 0.0431	0.7611	0.9848 ± 0.0179	0.9936

The results on DS-Cv2 demonstrate the robustness and generalization capabilities of our approach across different datasets. CNN-based models generally outperform classical machine learning classifiers, particularly when combined with ProtBERT and ProtBERT-BFD. Notably, the ProtBERT-BFD + CNN combination achieves the highest performance on the independent test set, with an MCC of 0.9492 and ROC AUC of 0.9968, underscoring the effectiveness of combining pre-trained language models with deep learning architectures for ion channel classification.

MembraneBERT shows strong performance with classical classifiers, often outperforming ProtBERT and ProtBERT-BFD. However, its performance with CNN is unexpectedly lower, particularly in terms of sensitivity on the independent test set, suggesting that MembraneBERT’s embeddings may have characteristics better suited to classical machine learning approaches.

Among classical classifiers, Random Forest (RF) and Logistic Regression (LR) show the most consistent performance across different PLMs, indicating their robustness to variations in input representations. SVM, on the other hand, exhibits high variability in performance, particularly with ProtBERT-BFD and MembraneBERT.

We observed some discrepancies between cross-validation and independent test performance, particularly for sensitivity. This suggests that while the models generally generalize well, there might be some overfitting or dataset-specific characteristics influencing the results. Notably, all models achieve very high specificity ( > 99 %) on the independent test set, indicating excellent performance in correctly identifying non-ion channel proteins.

The overall improved performance on DS-Cv2 compared to the original dataset (DS-C) can be attributed to several factors. First, DS-Cv2 contains significantly more samples, providing more diverse and representative training data. Second, the updated dataset incorporates the most recent protein annotations, potentially offering more accurate and refined information for classification. Lastly, the new dataset may provide a more balanced representation of different types of ion channels and membrane proteins, leading to improved generalization.

**Figure 8:**
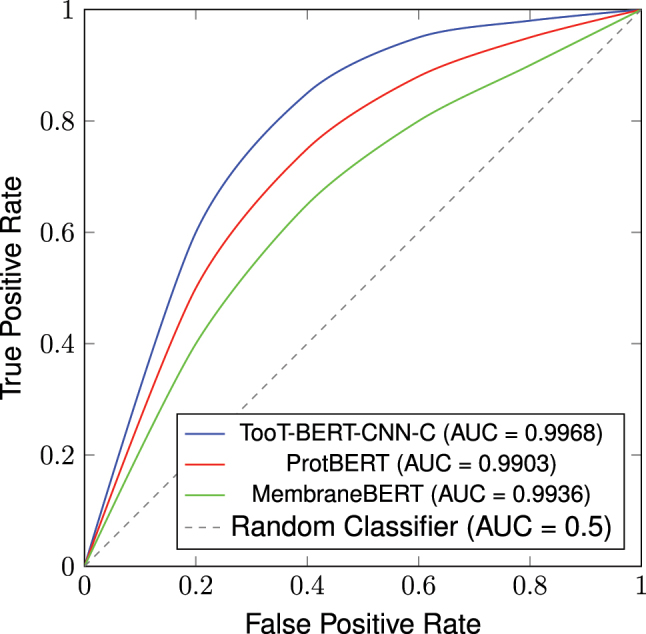
Stylized ROC curves comparing the performance of different models based on their AUC values.

The ROC curves illustrated in Figure 8 provide a visual comparison of the performance of our models in distinguishing ion channels from non-ion channels. All three models – TooT-BERT-CNN-C, ProtBERT, and MembraneBERT – demonstrate exceptional performance, with curves that hug the top-left corner of the plot, indicating high true positive rates even at low false positive rates. TooT-BERT-CNN-C, with an AUC of 0.9968, shows a slight but noticeable superiority over the other models, particularly in the lower false positive rate range. This suggests that TooT-BERT-CNN-C maintains high sensitivity without sacrificing specificity, a crucial characteristic for reliable ion channel classification. ProtBERT and MembraneBERT also show strong performance with AUCs of 0.9903 and 0.9936 respectively, but their curves fall slightly below that of TooT-BERT-CNN-C, especially in the early stages of the curve. All models significantly outperform the random classifier baseline (represented by the diagonal line), underscoring the effectiveness of our approach in leveraging protein language models for this classification task. The minimal differences between these high-performing models highlight the robustness of our methodology across different protein language model architectures, while also demonstrating the incremental improvement achieved by TooT-BERT-CNN-C.

### 3.5 Comparison to state-of-the-art

**Figure 9:**
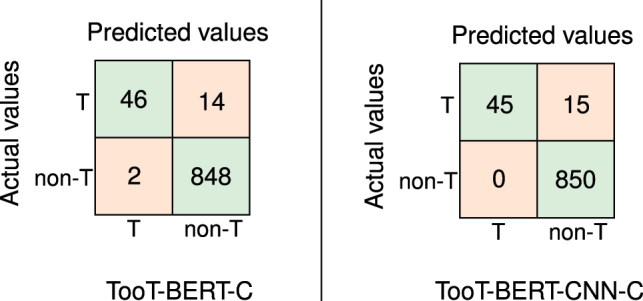
Confusion matrices for TooT-BERT-C and TooT-BERT-CNN-C. This figure presents confusion matrices for two approaches, TooT-BERT-C and TooT-BERT-CNN-C, used in the task of ion channel prediction.

Figure 9 presents confusion matrices for our binary classification models: TooT-BERT-C and TooT-BERT-CNN-C. TooT-BERT-C achieves 46 True Positives (TP), 848 True Negatives (TN), 2 False Positives (FP), and 14 False Negatives (FN). TooT-BERT-CNN-C slightly outperforms this with 45 TP, 850 TN, 0 FP, and 15 FN, primarily due to its increased TN and reduced FP counts.

Table 6 compares TooT-BERT-CNN-C with three established methodologies: DeepIon [15], MFPS_CNN [14], and TooT-BERT-C [37]. TooT-BERT-CNN-C consistently outperforms these approaches across most metrics. On the independent test set, it achieves the highest scores in accuracy (98.35 %), specificity (100 %), and MCC (0.86). In cross-validation, it leads in accuracy (99.39 %) and MCC (0.95), while matching the highest specificity (99.82 %).

**Table 6:** Comparative performance of TooT-BERT-CNN-C with state-of-the-art. This table compares the performance of TooT-BERT-CNN-C with state-of-the-art approaches on cross-validated and independent test sets using evaluation metrics such as sensitivity, specificity, accuracy, and MCC. The maximum value in each column is highlighted in boldface.

Method	Acc (%)		Sen (%)		Spc (%)		MCC	
	Ind.	CV	Ind.	CV	Ind.	CV	Ind.	CV
DeepIon [15]	86.53	87.05	68.33	89.20	87.72	84.89	0.37	0.75
MFPS_CNN [14]	94.60	96.50	**76.70**	**95.00**	95.80	98.00	0.62	0.93
TooT-BERT-C [37]	98.24	98.96	76.67	86.71	99.76	**99.82**	0.85	0.91
TooT-BERT-CNN-C	**98.35**	**99.39**	75.00	93.38	**100.00**	**99.82**	**0.86**	**0.95**

The superior performance of TooT-BERT-CNN-C can be attributed to its hybrid architecture, which combines BERT-based embeddings with a CNN classifier. This allows it to capture both context-sensitive sequence nuances and hierarchical data representations effectively.

A McNemar’s test comparing TooT-BERT-CNN-C and TooT-BERT-C yields a *p*-value of 0.0625, suggesting a statistically significant improvement in performance.

## 4 Conclusions

In this research, we have made significant strides in advancing the accurate classification of ion channels and non-ion channels, a challenge with profound implications for both biology and medicine. Building on our prior work with TooT-BERT-C, we extended our investigation to encompass a broader array of classical classifiers and introduced a Convolutional Neural Network for comparative assessment.

Our empirical findings substantiate that the newly proposed method, TooT-BERT-CNN-C, exceeds both the state-of-the-art and our preceding approach in performance metrics. On our original dataset, we observed a boost in the Matthews Correlation Coefficient from 0.8486 to 0.8584, along with an uptick in accuracy rates from 98.24 % to 98.35 %. Even more impressively, on our newly curated dataset DS-Cv2, the ProtBERT-BFD + CNN combination achieved an MCC of 0.9492 and ROC AUC of 0.9968 on the independent test set. These results not only affirm the efficacy of our methodology in discerning ion channels from non-ion channels but also underscore the promise of fusing pre-trained language models with deep learning architectures for this application. The superior performance on the larger, more recent DS-Cv2 dataset further validates the robustness and generalizability of our approach.

Looking ahead, several promising avenues for future work emerge from our findings. We envision exploring alternative pre-trained language models and deep learning architectures to potentially further improve performance. Additionally, investigating feature engineering techniques could provide deeper insights into the molecular characteristics that distinguish ion channels from other membrane proteins. The development of a web server stands out as a crucial next step, as it would make our tool more accessible to the broader scientific community. This would involve creating a user-friendly interface, implementing backend infrastructure, integrating database functionality, ensuring scalability, and implementing robust security measures. Furthermore, extending our approach to more fine-grained classification tasks, such as distinguishing between different types of ion channels, could enhance the utility of our tool. Integrating structural information, where available, also holds potential for enhancing prediction accuracy.

Based on our findings, we recommend that future research in this field focus on further exploring the synergy between protein language models and deep learning architectures, as this combination has shown exceptional promise in our study. Investigating the specific features learned by our models, particularly the CNN, could yield valuable insights into the molecular signatures of ion channels, potentially informing our understanding of their structure and function beyond mere classification.

In conclusion, our work represents a significant advancement in computational methods for ion channel classification. By leveraging the power of protein language models and deep learning, we have developed a highly accurate tool that could accelerate research in ion channel biology and potentially aid in drug discovery efforts targeting these crucial membrane proteins. The implementation of these recommendations could further enhance the utility and impact of this approach in the field of bioinformatics and molecular biology.
